# The Role MicroRNA-135a in Suppressing Tumor Growth in Kidney Cancer Through the Regulation of Phosphoprotein Phosphatase2A and the Activation of the AKT and ERK1/2 Signaling Pathways

**DOI:** 10.7150/jca.90756

**Published:** 2024-01-01

**Authors:** Kangning Wang, Hege Chen, Xiang Chen, Zesong Fang, Enhua Xiao, Qiuling Liao

**Affiliations:** 1 Department of Urology Surgery, Xiangya Hospital Central South University, Changsha Hunan Province, 410008, China.; 2 Department of Urology laboratory, Guangdong Medical University, Zhanjiang, Guangdong Province, 524001, China.; 3 Department of Radiology, The Second Xiangya Hospital of Central South University, Changsha 410011, Hunan, China.

**Keywords:** MiR-135a, PP2A, Akt, ERK1/2, Replication, Kidney cancer

## Abstract

**Background:** Kidney cancer is a frequently occurring malignant tumor in the urinary system, with rising morbidity and mortality rates in recent times. Developing new biomarkers and therapeutic targets is essential to improve the prognosis of patients affected by kidney cancer. In recent years, miRNAs' role in tumorigenesis and development has received growing attention. miRNAs constitute a group of small non-coding RNA molecules that regulate gene expression, affecting various biological processes, including cell proliferation, differentiation, and apoptosis. Of the many miRNAs, miR-135a plays a pivotal role in several cancers. Nevertheless, the precise mechanisms and functions concerning miR-135a in renal cancer remain incompletely understood. Therefore, this study aims to analyze the effects of miR-135a on renal cancer replication and migration and its possible mechanisms, and to provide new strategies for the diagnosis and treatment of renal cancer.

**Methods:** Renal cell lines (ACHN, A498) with stable hyperexpression of miR-135a and reduced expression of miR-135a were constructed by lentivirus packaging. The changes of replication, clone formation and migration ability of overexpressed miR-135a and overexpressed miR-135a in ACHN and A498 renal cell lines were detected. The possible mechanism of miR-135a affecting the replication of kidney cancer was analyzed by target gene prediction, double luciferase test, Western blotting and subcutaneous tumorigenicity assay in nude mice.

**Results:** Hyperexpression of miR-135a can inhibit kidney cancer replication, whereas miR-135a knockdown potentially enhances replication. However, neither hyperexpression nor knockdown of miR-135a affects the migration ability of kidney cancer cells. The protein expression of PP2A-B56-γ, PP2A-Cα and PP2A-Cβ in renal cell line decreased after hyperexpression of miR-135a, while the protein expression of PP2A-B56-γ, PP2A-Cα and PP2A-Cβ increased after knockdown of miR-135a. In addition, the protein expression of p-Akt and p-ERK1/2 proteins in kidney cancer cells after hyperexpression of miR-135a were down-regulated, while the protein expression of p-Akt and p-ERK1/2 were up-regulated in kidney cancer cells after knockdown of miR-135a. In subcutaneous tumor formation experiments in nude mice, tumor size within nude mice in the miR-135a group was significantly smaller than in the control group.

**Conclusion:** MiR-135a could suppress the replication of kidney cancer by modulating PP2A and AKT, ERK1/2 signaling pathways.

## Background

Kidney cancer (KC) ranks as the third most common malignant tumor in urinary system following bladder cancer and prostate cancer. More than 80% of KC are renal clear cell carcinoma, whose incidence is increasing year by year 1, 2. At present, the main treatment of renal cancer is surgery. Nevertheless, due to the lack of typical symptoms in the early stage, the diagnosis of renal cancer is in the middle and late stage. Among it, about 30% of the patients have spread when they are diagnosed, thus missing the best opportunity of radical surgery 3, 4. In addition, renal cancer is not responsive to radiotherapy and chemotherapy. This presents a challenge in treating patients with advanced renal cancer, as their prognosis is poor and survival rate low 5-7. Currently, surgical treatment remains the only effective option for curing renal cancer due to the lack of targeted drugs for this specific indication. Thus, it is of importance to reveal the molecular mechanism of KC and to diagnose and treat it early.

MiRNA is associated with tumor replication, migration and invasion 8, 9. Recent research has revealed that a variety of miRNA are abnormally expressed in KC programmed death spread 10-12. For instance, miR-135a is the 4th small RNA molecule with the ability to suppress cell replication 13. It can induce programmed death of renal cancerous cells and impede their replication 14, 15. However, the specific mechanism of its action is yet to be determined. Therefore, further study on the involvement of miR-135a in KC has certain practical significance for the diagnosis and treatment of KC, and may also enrich the etiology and molecular pathology theory of KC.

Phosphoprotein phosphatase2A (PP2A) is the main Ser/ Thrphosphoprotein phosphatasein eukaryotes, which can dephosphorize protein and play a regulatory role in the physiological processes of cell differentiation, metabolism, replication, programmed death and cell transformation 16, 17. Due to the diversity of the B family of PP2A, PP2A regulation is complex with numerous substrates. Therefore, the role of PP2A holoenzymes with varying regulatory subunits in tumorigenesis and development necessitates further research 18. Akt is a very important protein kinase in the cellular signaling network of PI3K/Akt 19. Extracellular signal-regulated kinase (ERK) is an important subfamily of mitogen-activated protein kinases. It belongs to tyrosine protein kinases which can only exert their activity after phosphorylation 20. The AKT and ERK1/2 signaling pathways have been thoroughly researched and demonstrated to have vital functions in the development and advancement of different tumors. They are recognized as important regulators of cancerous cell proliferation, survival, and invasion. Consequently, controlling the regulation of these two signaling pathways could have crucial implications in hindering tumor growth. Previous studies have revealed possible links between miR-135a and AKT and ERK1/2 signaling pathways. MiR-135a can regulate the activity of target genes associated with AKT and ERK1/2 signalling pathways, indirectly affecting them. Further investigation into how miR-135a interacts with these pathways could elucidate its mechanism in inhibiting KC growth. If miR-135a is proven effective in activating these signaling pathways to inhibit tumor growth, it could provide new possibilities for KC therapy. At present, there is no research available on the targeted regulation of PP2A by miR-135a in renal carcinoma, nor any studies conducted on the relationship between miR-135a and Akt or ERK1/2 signal pathways. Therefore, it is necessary to further explore the involvement of miR-135a in KC as well as its related mechanisms of acting with PP2A, Akt and ERK1/2 molecular signaling networks in KC.

## Materials and Methods

### Cell Lines and Cell Culture

ACHN and A498 cell lines were bought from Guangzhou Jennio Biological Technology Co., Ltd, and ACHN cell was cultured in High glucose DMEM medium supplemented with 10% FBS and 5% penicillin-streptomycin (PS) (100×). A498 cell was cultured in RPMI-1640 medium supplemented with 10% FBS and 5% PS (100×).

### MiRNA Transfection

The DNA fragment for miR-135a, control miRNA, and siRNAs were obtained from Gene Copoeia, Inc and inserted into lentiviral expression plasmids. The human renal cancer cell lines ACHN and A498 were cultured in appropriate media with 10% FBS supplementation for 24 hours before transfection. The transfection procedure involved adding miR-135a, miR-135a inhibitor, and control viruses to the culture medium. After transfection, cells were cultured in media supplemented with 10% FBS and 0.5 μg/mL puromycin for selection.

### Luciferase activity assay

This method was conducted in accordance with the manufacturer's protocol of Dual-Luciferase Reporter 1000 Assay System. 293T cells were plated in 24-well cell culture clusters (Corning Incorporated; Corning, NY, USA). Upon reaching 70% confluence, the cells were co-transfected with hsa-miR-135a/control miRNA along with the 3'-UTR fragments of PP2A-Cα, PP2A-Cβ, or PP2A-B56-γ. After 48 hours of transfection, the cells were collected for the assessment of firefly and Renilla luciferase activities. Renilla luciferase activities were utilized for normalization of transfection efficiency.

### QRT-PCR

Extraction of total RNA was conducted using AG RNAex Pro RNA extraction kit AG21102, followed by measurement of RNA purity and concentration via NanoDrop™ 2000 spectrophotometer. Synthesis of complementary DNA (cDNA) was conducted via Evo M-MLV Mix Kit with gDNA Clean for qPCR AG11728, and detected in the QuantStudio^TM^ 5 Real-time PCR instrument. Real-time quantitative PCR was conducted using the SYBR Green PremixPro TaqHS qPCR Kit (Rox Plus), with AG11718.U6 as the loading control genes and miR-135a, following the manufacturer's instructions outlined in Table 1. Gene expression was calculated by the 2-^ΔΔCt^ method.

### Western Blotting

Cells were lysed with RIPA buffer and total proteins were extracted. Western blotting was carried outusing standard procedures. The proteins underwent SDS-PAGE separation and subsequent transfer onto a PVDF membrane. The blotted membranes were incubated with anti-PP2A C subunit antibody, anti-PP2A-B56-γ antibody, anti-PP2A-Cα antibody, anti-PP2A-Cβ antibody, AKT antibody, P-AKT antibody, ERK1/2 antibody, P-ERK1/2 antibodies, oranti-β-tubulin antibody, respectively, and then probed with a secondary antibody (1:10000,), with β-tubulin as a loading control.

### Cell Replication (CCK-8)

Four separate cultures of cells were transfected with control miRNA, miR-135a, control miRNA inhibitor, and miR-135a inhibitor, and each culture was seeded in an individual 96-well cell culture cluster. The cultures were then maintained for a period of 5 days. To assess cell replication, the cell proliferation reagent CCK-8 was introduced into each well and incubated for an additional 1 hour. The absorbance at 450 nm was subsequently measured.

### Colony Assay

For the colony assay, a total of 1000 cells were plated in 60mm plastic dishes containing 3 mL of DMEM medium supplemented with 10% FBS. The dishes were then incubated at 37°C in a humidified atmosphere with 5% CO2. ACHN cells were cultured for three weeks, while A498 cells were cultured for two weeks. After the incubation period, the colonies were stained with CBB and counted. All studies were conducted with 3 replications.

### *In vitro* Scratch Assay

The cells were plated in a 12-well cell culture plate for *In vitro* scratch assays, cell monolayers were scratched with a sterile pipette tip after cells reached 100% confluence. The cell migrations were observed for up to 24h. All percent of wound closures were conducted with 3 replications, and per replicate was calculated for five randomly chosen fields.

### Effect of miRNA-135a on tumor growth in nude mice for subcutaneous tumorigenesis detection

The nude mice used in the experiment were purchased from China Hunan Slack Jingda Laboratory Animal Co., Ltd. Firstly, according to regulations, disinfect and prepare the breeding room and cage in advance, and then test nude mice for one week to adapt to the environment. The experiment was divided into a control group and a miRNA-135a group. The miRNA-135a group of nude mice injected miRNA-135a transfected cancer cell A498 subcutaneously into the nude mice; The control group injected control miRNA transfected cancer cell A498 subcutaneously into the nude mice. During this period, the growth status of nude mice was observed daily, and the size of subcutaneous tumors in nude mice was recorded using an electronic caliper. The weight of nude mice was recorded using an electronic scale, while the survival status of nude mice was also recorded. When the subcutaneous tumor of nude mice grows to 1.0-1.5 centimeters, the nude mice are euthanized using the neck removal method, and the tumor is carefully separated and measured in millimeters, accurate to two decimal places. Finally, calculate the volume of the tumor and perform relevant statistical analysis using the statistical software GraphPad Prism 9.0.

### Statistical Analysis

All statistical methods were conducted using GraphPad Prism 5.0 (GraphPad Software, Inc., USA), and the results were presented ad mean ± S.D. Statistical significance was considered for *P* < 0.05, while highly significant differences were indicated for *P* < 0.01 and *P* < 0.001.

## Results

### Construction of ACHN and A498 Renal Cell Lines with Stable Hyperexpression of MiR-135a, Reduced Expression of MiR-135a and Expression Independent Sequences

**Figure 1:**
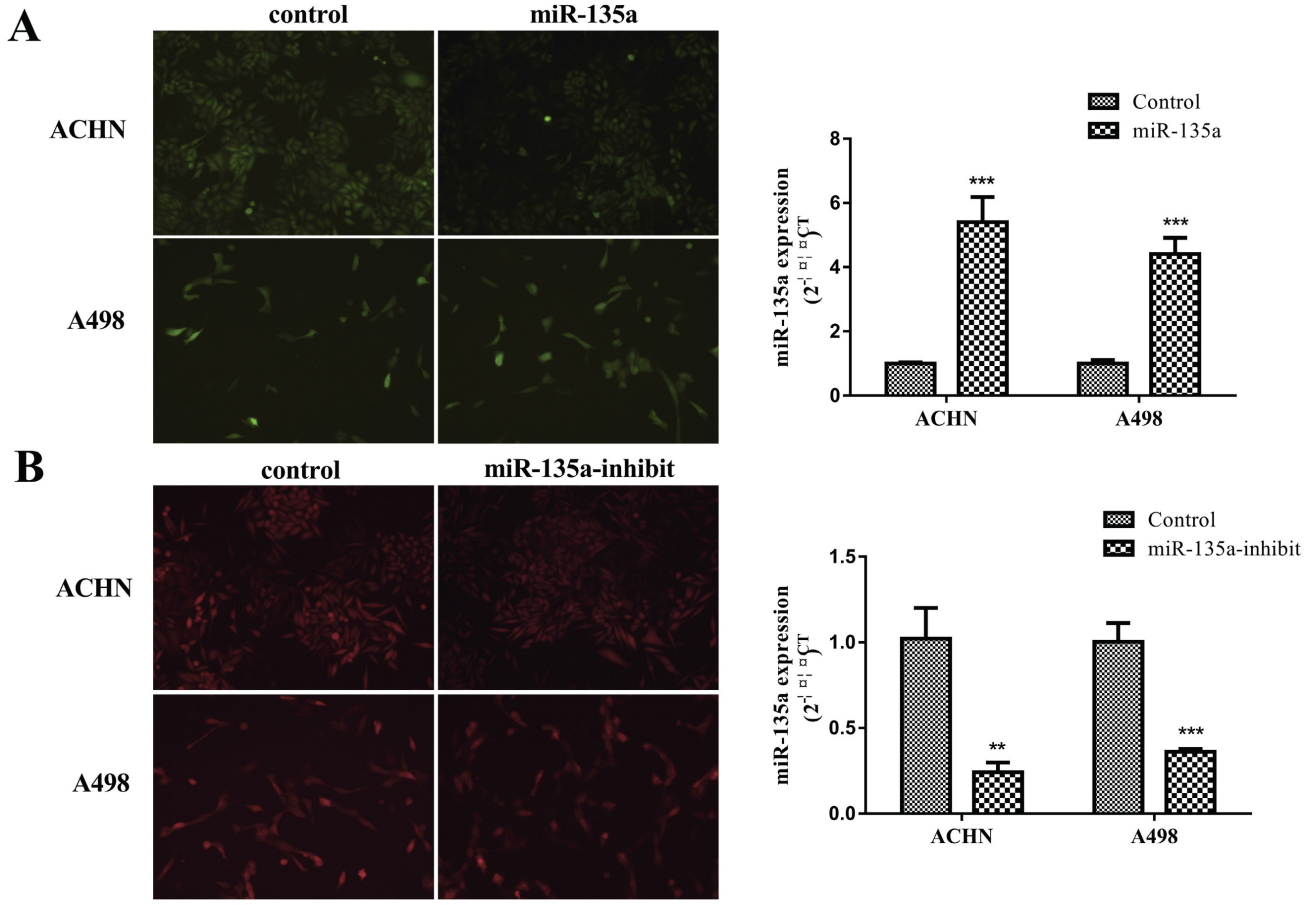
** Stable hyperexpression and reduced expression of miR-135a ACHN and A498 renal cancer cell lines.** A. Kidney cancer cell lines of ACHN and A498 hyperexpression miR-135a; B. Kidney cancer cell lines of ACHN and A498 underexpressing miR-135a.

MiR-135a plasmid contains green fluorescent protein gene, and miR-135a-suppress plasmid contains red fluorescent protein gene. Observation of fluorescence expression using an inverted fluorescence microscope allows for determination of plasmid infection and expression in KC. Purinomycin was utilized in screening for uninfected cells with the target gene, resulting in acquisition of ACHN and A498 KC cells with stable hyperexpression of miR-135a, reduced expression of miR-135a, and independent expression sequences. Total RNA was extracted, and then the expression of miR-135a was measured by fluorescence quantitative PCR in real time. The results showed that the expression of miR-135a in ACHN and A498 renal cancer cells containing lentivirus with hyperexpression of miR-135a was significantly greater than those in the control group (ACHN *P* < 0.001, A498 *P* < 0.001; Fig. 1A). It could be seen that more than 95% of KC cells expressed green fluorescent protein. However, the expression of miR-135a in ACHN and A498 cells infected with lentivirus incorporating the gene of interest of miR-135a suppressor decreased significantly (Fig. 1B). It can be seen that more than 95% of KC cells expressed red fluorescent protein. This indicated that the renal cell line with stable hyperexpression of miR-135a and reduced expression of miR-135a was successfully constructed.

### Hyperexpression of miR-135a could suppress the replication of KC, but had no obvious effect on the migration ability of KC

**Figure 2:**
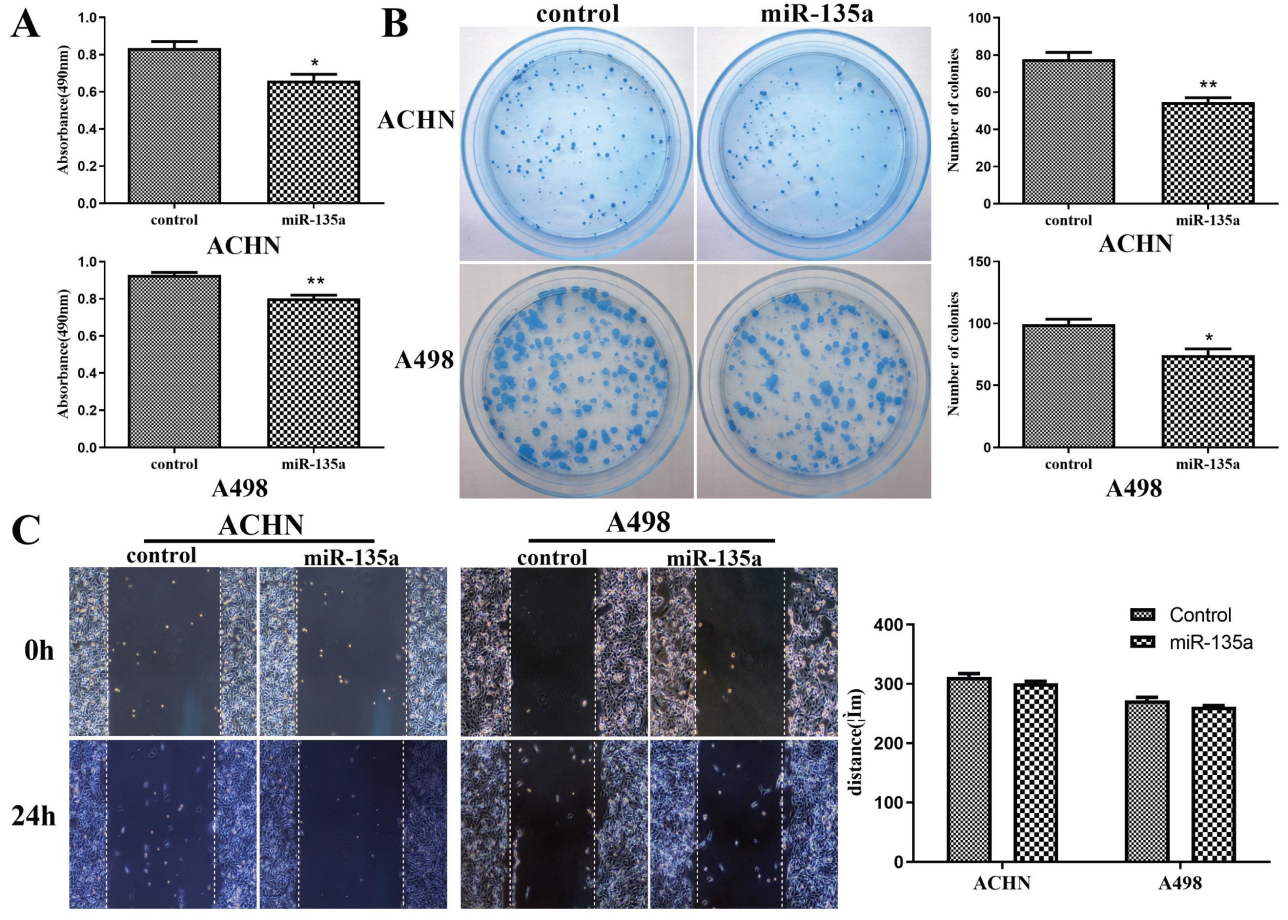
** Changes in the replication, clone formation ability and migration ability of kidney cancer cells after hyperexpression of miR-135a.** A. CCK-8 method to detect the changes of cell replicationability; B. clone formation test to detect the changes of cell clone formation ability; C. Cell division Scar assay for measuring cell migration capacity. *P < 0.05, **P < 0.01.

CCK-8 experiment revealed that the hyperexpression of miR-135a could significantly suppress the replication of KC cells ACHN and A498 (ACHN P < 0.05, A498 *P* < 0.01; Fig. 2A). However, the clone formation test showed that the number of cell clones formed after hyperexpression miR-135a was significantly lower compared to control group, and the results were consistent with the results of cell replication (Fig. 2B). The results from the cell scratch test exhibited that there was no significant difference in the migration distance of ACHN and A498 cells with hyperexpression of miR-135a after 24 hours in comparison to the control group, as shown in Fig. 2C. Therefore, these results suggest that miR-135a hyperexpression has the capability to hinder KC cell growth, but does not significantly affect their migration ability.

### Reduced Expression of MiR-135a Could Promote the Replication of KC, But Had No Obvious Effect on The Migration Ability of KC

**Figure 3:**
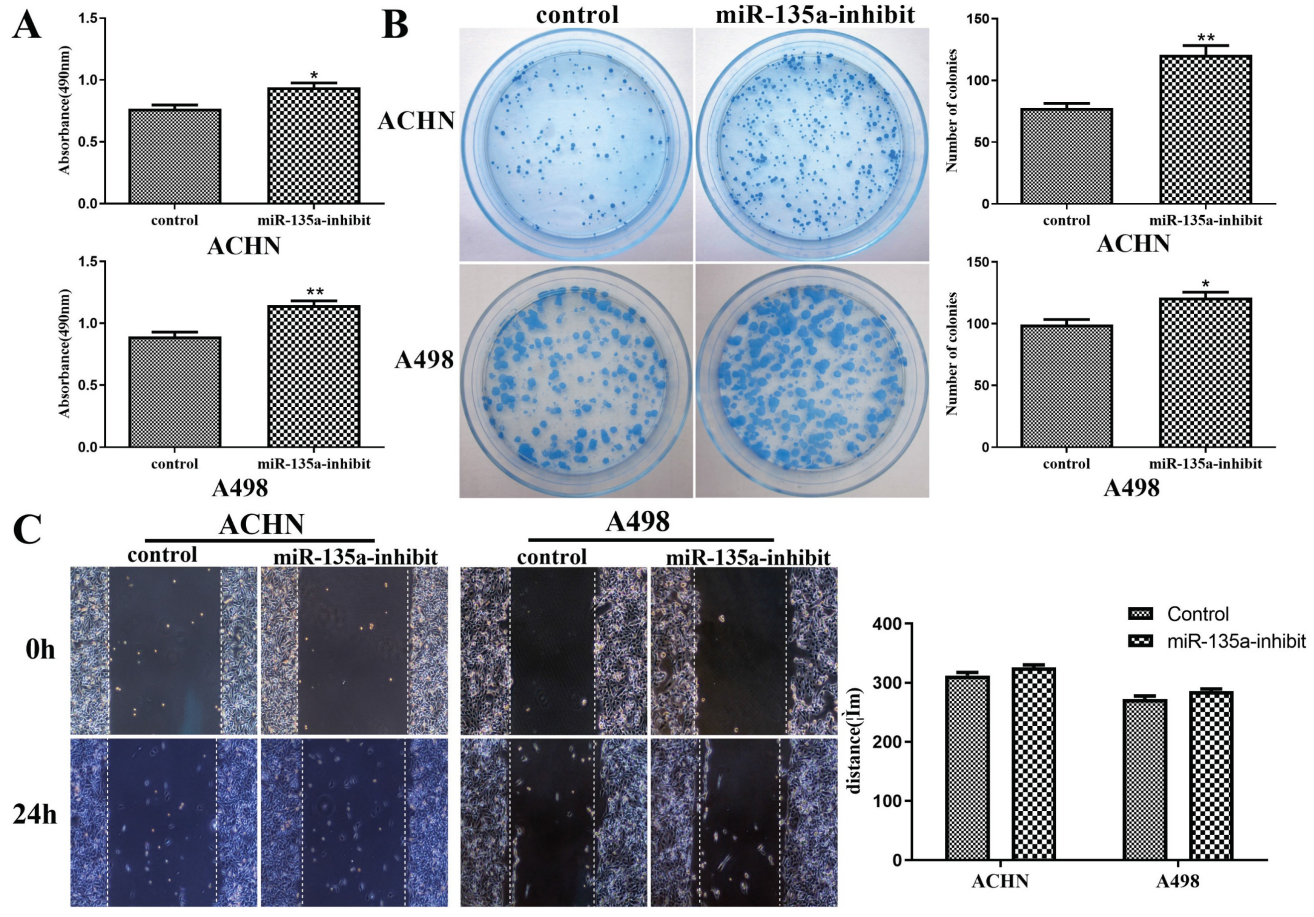
** Changes in replication, clone formation and migration ability of renal cancer cells after reduced expression of miR-135a.** A. CCK-8 assay; B. clone formation assay; C. Cell division Scar assay. *P < 0.05, **P < 0.01.

The results of CCK-8 experiment showed that the replication ability of ACHN and A498 renal cancer cells was enhanced after knockdown of miR-135a expression (Fig. 3A). At the same time, its clone forming ability was also improved (Fig. 3B).

The results of cell scratch test revealed that the knockdown of miR-135a had no obvious impact on the migration ability of ACHN and A498 cells. After 24 hours, the migration distance of ACHN and A498 with reduced expression of miR-135a showed no difference from the control group (Fig. 3C). This suggested that reduced expression of miR-135a could promote the growth of KC, but exert no impact on the migration ability of KC.

### MiR-135a Regulated Cell Replication Through PP2A-B56-γ, PP2A-Cα and PP2A-Cβ

**Figure 4:**
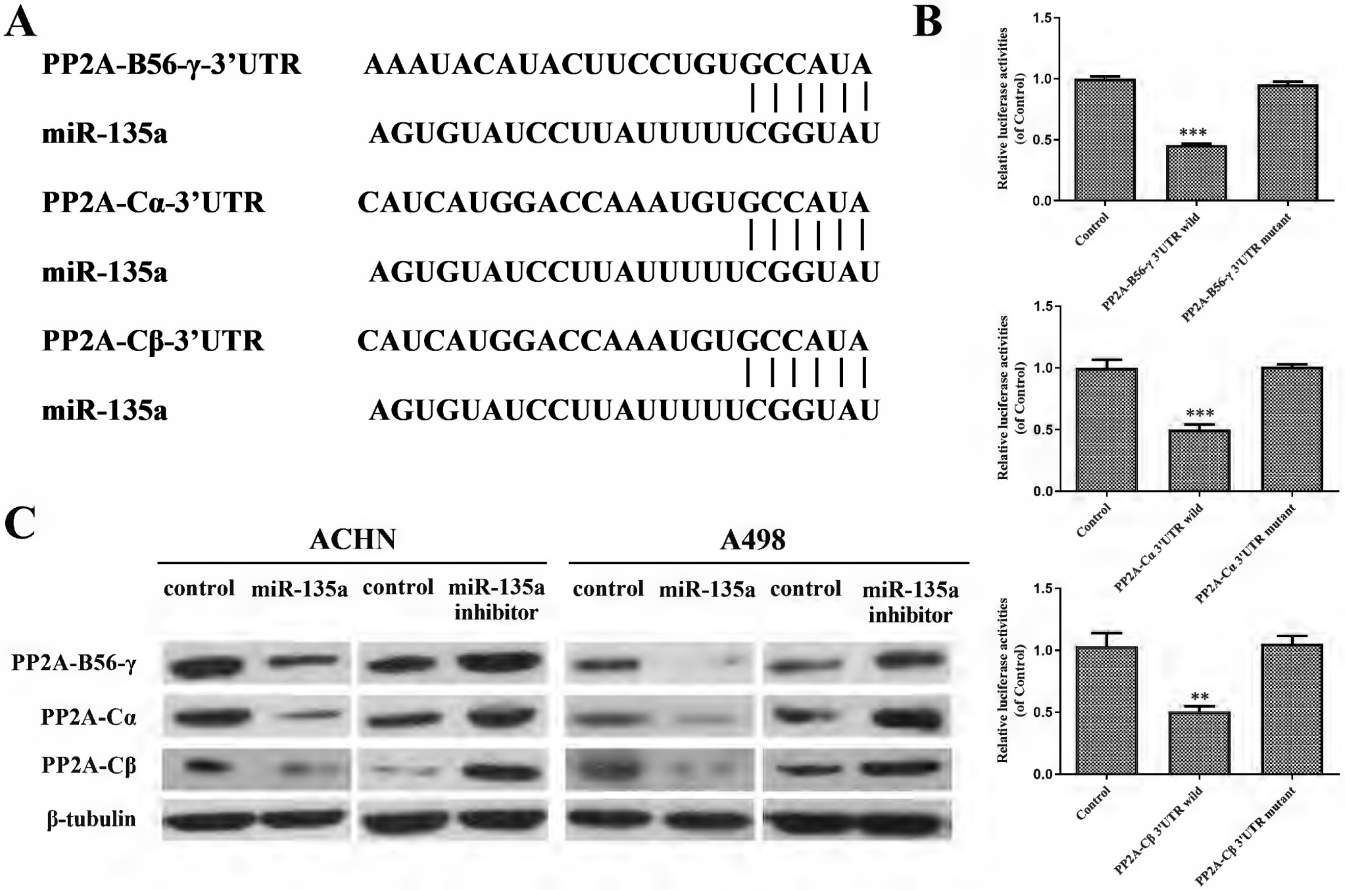
** miR-135a targets PP2A-B56-γ, PP2A-Cα and PP2A-CβA.** A. miR-135a is linked to the 3'UTR of target genes PP2A-B56-γ, PP2A-Cα and PP2A-Cβ; B. miR Luciferase assay results between -135a and wild-type/mutant PP2A-B56-γ, PP2A-Cα and PP2A-Cβ 3'UTR; C. PP2A-B56-γ, PP2A-Cα after hyperexpression/knockdown of miR-135a and PP2A-Cβ protein expression level changes. *P < 0.05, **P < 0.01, ***P < 0.001.

Target Scan predicted the subordinate genes of miR-135a and found that PP2A-B56-γ, PP2A-Cα and PP2A-Cβ might be the target genes of miR-135a (Fig. 4A). The result of double luciferase test showed that miR-135a could bind to wild-type pp2a-b56-γ-3' UTR in a targeted way, and its fluorescence expression decreased (*P* < 0.001; Fig. 4B). Furthermore, it can specifically bind to the wild-type PP2A-C α-3' UTR and PP2A-C β-3' UTR, resulting in reduced levels of fluorescence expression (PP2A-Cα *P* < 0.001, PP2A-C β *P* < 0.01; Fig. 4B). However, miR-135a had no effect on the 3'UTR of mutant PP2A-B56-γ, PP2A-Cα and PP2A-Cβ, and its fluorescence expression remained basically unchanged (*P* > 0.05; Fig. 4B). This indicated that PP2A-B56-γ, PP2A-Cα and PP2A-Cβ were the direct targets of miR-135a. Western blotting showed (Fig. 4C) that the expression of PP2A-B56-γ, PP2A-Cα and PP2A-Cβ were down-regulated in KC after hyperexpression of miR-135a. The protein expression of PP2A-B56-γ, PP2A-Cα and PP2A-Cβ were all up-regulated in KC cells after knockdown of miR-135a. This suggests that hyperexpression of miR-135a might suppress the growth of KC by suppressing the expression of modulating genes PP2A-B56-γ, PP2A-Cα and PP2A-Cβ.

### MiR-135a Might Suppress the Replication of KC by Affecting Akt and ERK1/2 Pathways

**Figure 5:**
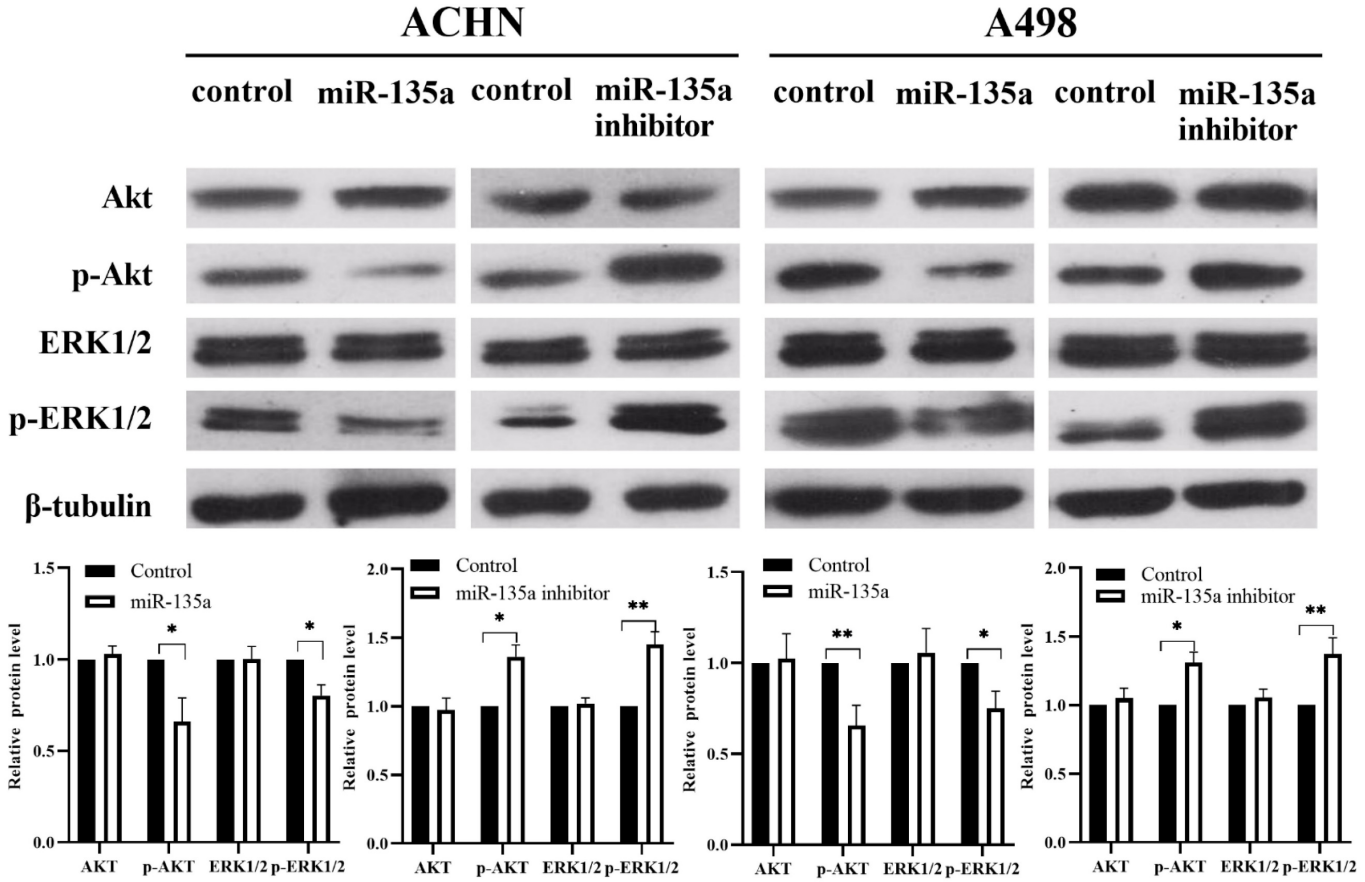
** Changes in Akt, p-Akt, ERK1/2, p-ERK1/2 expression levels after hyperexpression/knockdown of miR-135a.** Data are presented as mean ± SD (N = 3). *P < 0.05, **P < 0.01.

Our previous experiments have shown that the expression of miR-135a can suppress the replication of KC by down-regulating the expression of PP2A series proteins. At the same time, it is found that Akt and ERK1/2 may also be involved in the related mechanism of miR-135a in KC by consulting related literature and Western blotting. Western blotting showed (Fig. 5) that the expression of p-Akt and p-ERK1/2 were down-regulated in KC after hyperexpression of miR-135a, but the total level of Akt and ERK1/2 had no obvious change. The levels of p-Akt and p-ERK1/2 were found to be upregulated in KC cells following the knockdown of miR-135a. However, the total levels of Akt and ERK1/2 remained unchanged. These findings suggest that miR-135a overexpression may hinder the replication of KC by suppressing the phosphorylation of Akt and ERK1/2.

### Results of subcutaneous tumors in nude mice

**Figure 6:**
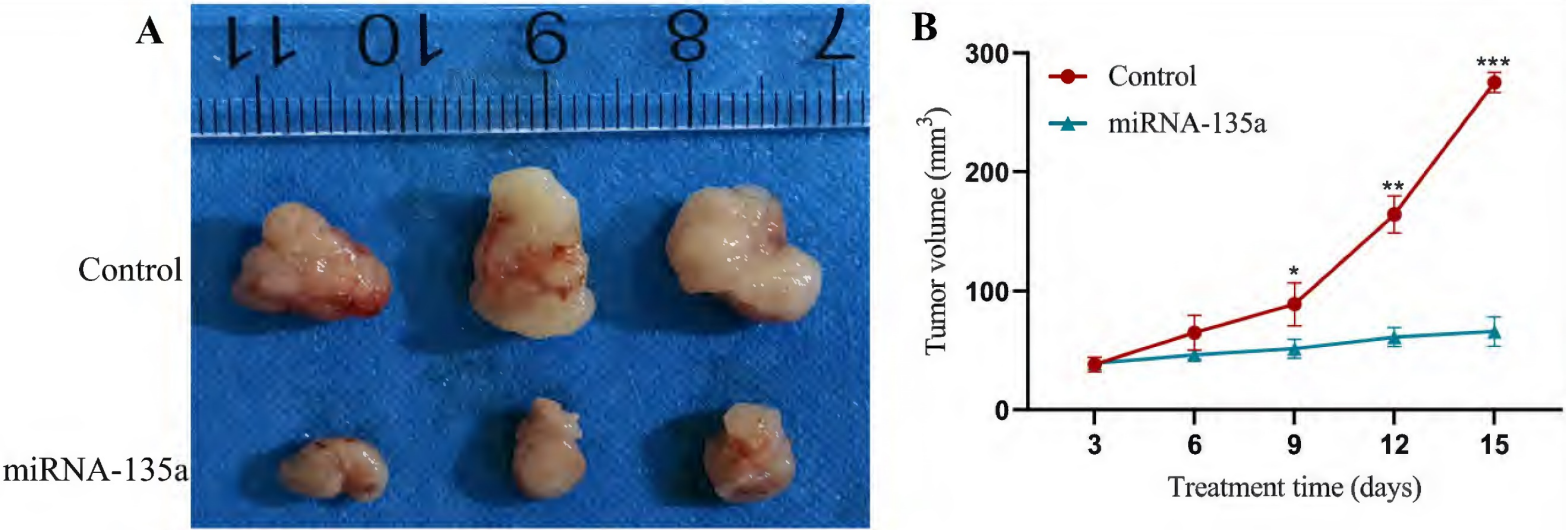
** Experimental results of subcutaneous tumors in nude mice of miRNA-135a group/control group.** A. Tumors formed after nude mouse tumor formation model; B. Tumor growth curve. *P < 0.05, **P < 0.01, ***P < 0.001.

Previous experiments have shown that miR-135a may inhibit KC replication by affecting the PP2A, Akt and ERK1/2 pathways. This experiment selected nude mice for subcutaneous tumorigenesis to test the effect of miRNA-135a on tumor growth, in order to verify the inhibitory effect of high expression of miR-135a on KC growth. After 15 days of the experiment, the tumor size in the miR-135a group of nude mice was significantly smaller than that in the control group. The results showed that the growth of KC *in vivo* could be significantly inhibited by overexpression of miRNA-135a (Fig. 6).

## Discussion

MiRNA participates in tumor cell replication, migration, invasion and programmed death by degrading target mRNA and suppressing protein translation through complementing the sequence of messenger RNA 21, 22. At the same time, miRNA is involved in key processes contributing to the initiation and advancement of tumors 23, 24. Although the research reports on the link between miRNA and various tumors are constantly emerging, there is still relatively few research on renal cancer. This topic mainly studies the link between miR-135a and KC. Several studies have explored the expression of miR-135a in KC and have found its expression to be lower in KC than in normal tissues 14. Furthermore, miR-135a has been found to have varying expression and functions among different types of tumors, indicating its significant role 25-28.

This study constructed ACHN and A498 renal cell lines with stable hyperexpression and low-expression of miR-135a by lentiviral infection. Further experiments confirmed that hyperexpression of miR-135a led to decreased replication ability in renal cell lines, whereas knock-down of miR-135a resulted in an obvious increase in replication ability. In subsequent subcutaneous tumor formation experiments in nude mice, the tumor size of nude mice in the miRNA-135a group was significantly smaller than that of the control group, which also verified the above observation. Additionally, the study indicated an inverse association between expression of miR-135a and PP2A gene, with a certain correlation to tumor initiation and advancement. The present study indicated that miR-135a could suppress the replication of KC by modulating PP2A gene. This is consistent with the conclusion of Japanese scholar Yamada et al 14. that miR-135a shows reduced expression in KC (caki2 and A498) and can suppress the replication of KC by modulating the expression of oncogene c-MYC and the process of cell cycle. In addition, either hyperexpression or reduced expression of miR-135a exerted no impact on the migration ability of ACHN and A498 cells (*P*<0.05). The specific mechanism needs to be confirmed by further experiments. Previous studies have shown that miR-135a regulates cancer cell replication and programmed death and also contributes to tumor invasion and spread. In 2020, Deng et al. 29 discovered that miR-135a can inhibit NSCLC cell replication, invasion, and spread through down-regulation of RAB1B and the RAS pathway, effectively suppressing the progression and spread of NSCLC. Studies have also shown that miR-135a can suppress the migration of gastric tumor cells by modulating the NF-κB pathway mediated by TRAF5 30. This difference may be caused by different tissues.

It is predicted that PP2A-B56-γ, PP2A-Cα and PP2A-Cβ were the targeted regulatory genes of miR-135a by Target Scan target gene prediction software, and verified that miR-135a could modulate the mRNA3'UTR of PP2A-B56-γ, PP2A-Cα and PP2A-Cβ by double luciferase test. Western blot analysis indicated that up-regulation of miR-135a suppresses the replication and development of KC by reducing PP2A gene expression. Conversely, the detection results of KC after down-regulation of miR-135a revealed an increase in the PP2A-B56-γ, PP2A-Cα, and PP2A-Cβ proteins. These results suggest that miR-135a regulates the PP2A series of target genes through post-transcriptional translation suppression. The present study innovatively revealed that PP2A could be modulated by miR-135a and played the role of oncogene in renal carcinoma. However, based on the prediction of target genes by bioinformatics, PP2A may be regulated by multiple miRNA other than miR-135a in renal cancer. For example, miR-183 can also regulate renal carcinoma 31 and promote cell growth, invasion, and spread by down-regulating PP2A, which has an anti-cancer effect in this study. This may be due to the complexity of the PP2A gene B family, which results in a diverse range of PP2A complexes and substrates, as well as different or even contradictory roles in the regulation of various genes. Therefore, the role of PP2A holoenzyme composed of different regulatory subunits in the initiation and advancement of needs further research and confirmation. In addition, the protein expression of phosphorylated Akt and ERK1/2 in KC cells after hyperexpression of miR-135a decreased obviously, while that of phosphorylated Akt and ERK1/2 was significantly up-regulated in KC cells after knockdown of miR-135a. This study demonstrates that overexpression of miR-135a may inhibit the replication of KC cells by suppressing the phosphorylation of Akt and ERK1/2. However, further research is needed to confirm the specific upstream activating proteins or downstream signaling proteins targeted by miR-135a. Additionally, the Akt and ERK1/2 signaling pathway could be one of the mechanisms through which miR-135a inhibits tumor cell replication. Further research into the mechanism by which miR-135a suppresses KC replication is crucial for understanding the self-regulatory replication process of KC and identifying potential therapeutic targets.

## Conclusion

Taken together, miR-135a can inhibit KC replication and tumor progression. Reducing endogenous miR-135a expression can increase KC replication. Moreover, this study showed that miR-135a can suppress the growth of kidney tumor cells through down-regulation of target genes PP2A-B56-γ, PP2A-Cα, and PP2A-Cβ. Additionally, miR-135a affects the AKT and ERK1/2 signaling pathways.

## Figures and Tables

**Table 1:** PCR primer sequences

	Primer sequence (5'- 3')
miR-135a	F: 5'-CCTCGCTGTTCTCTATGGCT -3'
	R: 5'-GAGTGAGCAGTAGAATCACAT -3'
U6	F: 5'- CGAATTTGCGTGTCATCCTT-3'
	R: 5'- CGAATTTGCGTGTCATCCTT-3'

Note: R: Reverse Sequence F: Forward Sequence
